# Isolated Post-Traumatic Radial Head Dislocation, A Rare and Easily Missed Injury-A Case Report

**DOI:** 10.5704/MOJ.1303.003

**Published:** 2013-03

**Authors:** V Gupta, ZS Kundu, SS Sangwan, D Lamba

**Affiliations:** Department of Orthopaedics, Pt. B.D.Sharma Post Graduate Institute of Medical Sciences, Rohtak, India; Department of Orthopaedics, Pt. B.D.Sharma Post Graduate Institute of Medical Sciences, Rohtak, India; Department of Orthopaedics, Pt. B.D.Sharma Post Graduate Institute of Medical Sciences, Rohtak, India; Department of Orthopaedics, Pt. B.D.Sharma Post Graduate Institute of Medical Sciences, Rohtak, India

## Abstract

Dislocation of the head of the radius may be either congenital, an isolated injury or more commonly part of a complex injury to the elbow such as the Monteggia fracturedislocation. Isolated traumatic radial head dislocation without associated injuries in children is a rare and easily missed condition. We report such a case in a 7-year-old boy without any associated injuries or co-morbid conditions. Initially the diagnosis was missed, and 6 weeks later open reduction was performed with annular ligament reconstruction surgery. At the one-year follow up, the patient had returned to most normal activities, showing only slight terminal restriction of pronation. We discuss the injury mechanism and management for the Monteggia fracturedislocation and review the available literature.

**Key Words:**

radial head dislocation, traumatic, Monteggia fracturedislocation

## Introduction

The radial head may be congenitally dislocated in isolation or in conjunction with other congenital abnormalities such as in diaphysial aclasis and dyschondroplasia, Ehlers-Danlos syndrome, nail-patella syndrome, trisomy 8, and achondroplasia. Traumatic dislocation of the radial head occurs most frequently in adults as part of a high-force injury. In children, radial head dislocations are usually complicated by complete elbow dislocations or fractures, as in the Monteggia complex^1^. Isolated traumatic dislocation of the radial head is extremely rare and in fact, frequently missed on initial evaluation. The rarity of these dislocations is evident by the lack of their description in the literature. Radial head dislocations are easily missed on radiographs and, therefore, require a high index of suspicion^2^. Undiagnosed chronic radial dislocations can result in poor outcomes with limited function and chronic pain. Elbow joint stability depends upon joint congruity and reduction of the radial head is very important for normal elbow function. Patients with radial head dislocation often have minimal pain and reasonable function, but are affected by increasing deformity and decreasing range of motion, eventually necessitating treatment.

The untreated radial head dislocation presents a diagnostic dilemma; further, stable open reduction requires annular ligament reconstruction to avoid repeated dislocation. The present report describes the characteristics of an untreated isolated radial head dislocation, its possible mechanism of injury and subsequent management.

## Case Report

**Fig. 1a & 1b:**
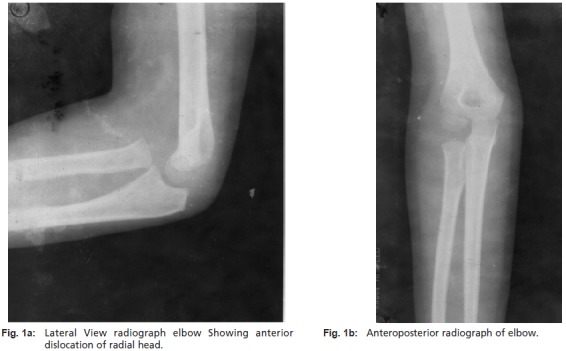
: (1a)Lateral View radiograph elbow Showing anterior dislocation of radial head. (1b) Anteroposterior radiograph of elbow.

While playing in a park, a 7-year-old male child fell on the outstretched left hand. He presented to the emergency department in a primary care centre with pain and swelling around the left elbow and reported difficulty in performing routine activities. At that time, the patient was treated for soft tissue injury around elbow with a Plaster of Paris back slab for 2 weeks. Six weeks after the injury, this patient was referred to us for persistent pain and restricted movement in the left elbow. Physical examination of the left elbow revealed normal skin with notable lateral fullness. Soft tissue anterolateral to the radial head was tender and there was restriction of supination and pronation. The elbow had full extension but terminal restriction of flexion. There was no joint effusion or evidence of posterior interosseus nerve palsy. There was no history of previous dislocations or pathology or joint laxity. Radiological examination was not suggestive of congenital dislocation of radial head. The injury did not meet the criteria for congenital dislocation of radial head as defined by McFarland and Mardem-Bey and Ger (Table I) 3. Radiographs showed an anterior dislocation of the radial head with no associated radial or ulnar fractures or disruption of the distal radio-ulnar joint. There was no evidence of ipsilateral ulnar bowing and the “ulnar bow sign” was negative (Figure 1).

**Fig. 2:**
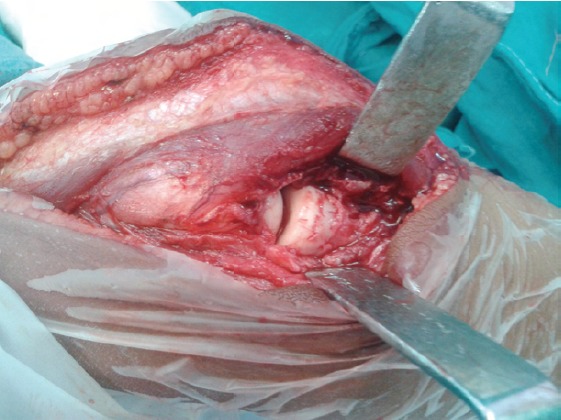
: Intraoperative photo after removal of fibrous tissue and relocation of radial head at anatomical position.

**Fig. 3a & 3b:**
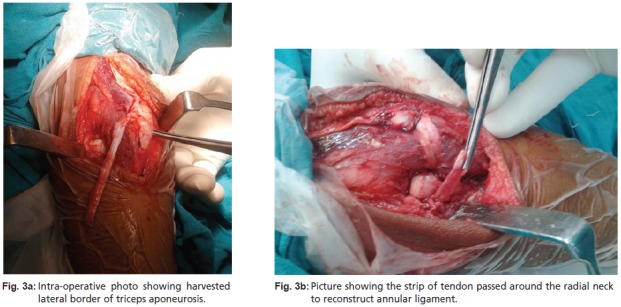
: (3a)Intra-operative photo showing harvested lateral border of triceps aponeurosis. (3b)Picture showing the strip of tendon passed around the radial neck to reconstruct annular ligament.

**Fig. 4a & 4b:**
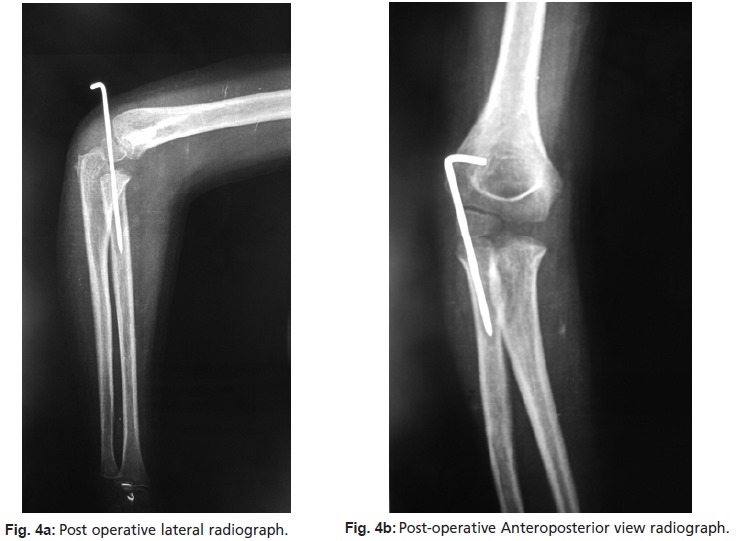
: 4(a)Post operative lateral radiograph. (4b)Post-operative Anteroposterior view radiograph.

As expected, closed reduction was not successful in this 6- week-old neglected dislocation. Open reduction under general anaesthesia was planned without further delay. Using a Boyd approach posterolateral skin incision to expose the radial head, we exposed the proximal radio-ulnar joint and found the annular ligament with much fibrosis and complete disruption. Button-holing of the annular ligament kept the radial head dislocated. Meniscus-shaped scar tissue around the radio-humeral and proximal radio-ulnar joints was completely excised to facilitate repositioning of the radial head using direct digital pressure, though at this stage the position was difficult to maintain. We removed capsular adhesions from the radial head and cleaned the radial notch of the ulna. Correct position was ascertained by observing the position of the radial head in the radio-capitellar joint and directly visualising the radio-ulnar joint (Figure 2). The articular surfaces of the radial head and capitellum were not damaged and there were no osteochondral fragments. Next, we dissected a 6-7 cm strip of the lateral border of triceps aponeurosis distally, carefully elevating the periosteum from the proximal ulna down to the level of the radial neck, taking care to preserve its attachment to the olecranon The strip of tendon was then passed around the radial neck, brought back and sutured to itself to reconstruct the annular ligament using a modification of the Bell-Tawse technique (Figure 3). The reduction was secured with 2mm K-wire passed through the posterior aspect of the capitellum into the radial head and neck with the elbow at 90 degrees and the forearm in supination. A post-operative radiograph showed acceptable congruent reduction (Figure 4). Postoperatively, a long arm plaster-of-paris cast was applied with the forearm in full supination. Forearm rotation in supination tightens the interosseous membrane and further stabilizes reduction.

After 3 weeks, the K-wire was removed and after 6 weeks, the cast was removed. Active exercises were initiated and the patient was instructed to report for regular follow up. At the one-year follow up, the patient had returned to his almost normal activities having almost full range of motion with slight terminal restriction of pronation.

## Discussion

Diagnosis of dislocation of the radial head can be easily missed because history is often vague, clinical findings are inconclusive and radiological features are relatively subtle. Careful radiological examination is the key to diagnosis. Isolated radial head dislocations caused by remote trauma and with no apparent lesion of the ulna have been mistaken for congenital radial head dislocations^3^. Lincoln and Mubarak described subtle anterior bowing of the shaft of the ulna as the ulnar bow sign. They suggested that the term isolated radial head dislocation was a misnomer and that these were actually variants of type I Monteggia injuries^1^,^2^. Although the absence of concomitant ulnar fracture is in part due to ulnar plasticity in children, Hudson et al. failed to find any ulnar bowing or periosteal reaction suggestive of fracture in their series of isolated radial head dislocation^4^. Radial head dislocation may occur secondary to rupture of a weak annular ligament even without significant ulnar trauma.

Although the exact mechanism of injury in dislocation of radial head is not clear, most often these injuries are associated with sporting activities and fall on an outstretched hand. The most common mechanism of injury involves a fall on an outstretched hand with a pronated forearm, a fully extended elbow and an additional varus strain applied to the ipsilateral elbow. In cadaveric specimens, anterior dislocations occurred with the forearm in extreme supination, by completely severing the anterior capsule and annular ligament, and applying force in an anterior direction to the posterior aspect of the radial head1. Tearing of the upper portion of interosseous membrane sometimes occurred before dislocation could be achieved.

Surgical correction is justified in irreducible and neglected/missed anterior dislocation of the radial head in children. Chronic radial head dislocation may lead to increasing valgus deformity of the elbow with subsequent ulnar or radial nerve dysfunction, restriction of flexion due to obstruction by radial head and consequently loss of function due to stiffness and instability^1^.

The present case involved anterior dislocation of the radial head in a 7-year-old male child. The child fell while running with his entire weight put on his outstretched left hand with his forearm pronated and elbow extended. With such hyperextension of the elbow, the radial head is at risk of anterior displacement through the annular ligament. Further, with a fall on an outstretched hand, force is transmitted down the shaft of radius and the annular ligament is disrupted anteriorly resulting in isolated radial head dislocation^4^. Button holing of annular ligament kept the radial head dislocated. Over time, the ligament contracts, fibroses, and intertwines with the radio-capitellar joint. In such situations, closed manipulation is unlikely to succeed more, resulting in a neglected dislocation. Therefore, in these cases, open reduction should be performed right away, as attempts at closed reduction will only serve to increase chondral damage or neural damage. As surgical repair of the dislocation is delayed, a more extensive surgical procedure is likely to be necessary to achieve a successful result.

For isolated traumatic radial head dislocation with accompanying annular ligament injury in children, surgery should be the treatment of choice and should include open reduction and annular ligament reconstruction surgery. As the role of annular ligament reconstruction in maintaining radial head reduction has been critically analysed, authors have advocated its use in every case that requires open surgery on the radio-capitellar joint^5^. Reconstruction involves harvesting a fascial slip from the triceps aponeurosis or the forearm fascia and creating a loop around the radial neck. This fascial slip acts both as a dynamic and static stabilizer, thereby preventing radial head subluxation.

Boyd used a slip from the extensor aponeurosis, Bell-Tawse used the central slip of triceps fascia and Lloyd-Roberts used the lateral slip but attached it distally^1^. Triceps aponeurosis is usually for reconstruction of the annular ligament, mainly because it is located close to the operative incision and involves less surgical trauma and a shorter operative time compared to use of other tissue; aponeurosis is tough and thick with rigid fixation and low risk of re-dislocation. Seel and Peterson suggested that the age of the patient and the duration of the dislocation are not important consideration in treatment choice for this injury. Their criteria for choosing surgical repair were the presence of a normal concave radial head articular surface and normal shape and contour of the ulna and radius.

Some authors recommend that, in addition to open reduction of the radial head and annular ligament reconstruction, angulation and elongation of the ulna by osteotomy are often necessary to maintain reduction of a chronically dislocated radial head. However, ligament reconstruction alone may facilitate radial head stability when forearm alignment is normal. Obviously, surgical procedures should be kept to a minimum but the orthopaedist must perform necessary procedures to obtain stable reduction of the elbow.

In conclusion, isolated post-traumatic radial head dislocation is uncommon and it is important that we recognize signs and symptoms, unusual mechanism of injury, and optimal management for this injury.

**Table I:**
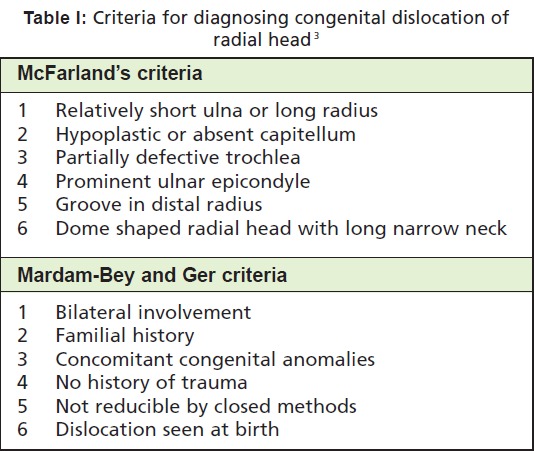
: Criteria for diagnosing congenital dislocation of radial head 3

## References

[1] Stans AA, Heinrich SD (2006). Dislocations of the elbow. In: Beaty JH and Kasser JR editors. Rockwood And Green’s Fracture in
children.

[2] Lincoln TL, Mubarak SJ (1994). "Isolated" traumatic radial-head dislocation. J Pediatr Orthop.

[3] McFarland B (1936). Congenital dislocation of head of radius. Br J Surg.

[4] Stanley D (1986). Isolated traumatic anterior dislocation of the radial head-a mechanism of injury in children. Injury.

[5] Li Z, He Y, Zhong G, Huang F (2011). Research progress in repair and reconstruction of isolated traumatic radial head dislocation with
annular ligament injury in children. Zhongguo Xiu Fu Chong Jian Wai Ke Za Zhi.

